# Characterization of Balance Problems and Rehabilitation Needs of Patients with Ménière’s Disease

**DOI:** 10.3390/audiolres12010003

**Published:** 2022-01-05

**Authors:** Ilmari Pyykkö, Nora Pyykkö, Jing Zou, Vinaya Manchaiah

**Affiliations:** 1 Hearing and Balance Research Unit, Field of Otolaryngology, Faculty of Medicine and Health Technology, Tampere University, 33100 Tampere, Finland; ilmari.pyykko@tuni.fi (I.P.); jing.zou@tuni.fi (J.Z.); 2 Faculty of Medicine, University of Tartu, 50090 Tartu, Estonia; nora.pyykko@gmail.com; 3 Department of Otolaryngology-Head and Neck Surgery, Center for Otolaryngology-Head & Neck Surgery of the Chinese PLA, Changhai Hospital, Second Military Medical University, Shanghai 200433, China; 4 Department of Otolaryngology-Head and Neck Surgery, University of Colorado School of Medicine, Aurora, CO 80045, USA; 5 UCHealth Hearing and Balance, University of Colorado Hospital, Aurora, CO 80045, USA; 6 Virtual Hearing La, Collaborative Initiative between University of Colorado School of Medicine and University of Pretoria, Aurora, CO 80045, USA; 7 Department of Speech-Language Pathology and Audiology, University of Pretoria, Gauteng 0002, South Africa; 8 Department of Speech and Hearing, School of Allied Health Sciences, Manipal University, Manipal 576104, India

**Keywords:** Ménière’s disease, Mal de Debarquement, balance problems, swaying, rocking, tripping

## Abstract

Background: To explore and characterize balance problems in subjects with Ménière’s disease (MD). Methods: A total of 539 people with MD with a mean age of 61.9 years, mean disease history of 15.6 years, and 79.5% females were recruited. The online questionnaire, consisting of 39 questions, including both structured and open-ended questions, focused on symptoms of MD, balance problems, impacts of the complaints, and quality of life (QoL). Results: After hearing loss (58%) and tinnitus (50%), balance problems (44%) were among the most commonly reported MD complaints, even higher than the impact of vertigo (40%). However, only 22% reported that those balance problems made obvious impacts in their daily lives. The most common balance problem that significantly reduced QoL was tripping (34%). Swaying (25%) had a limited impact on QoL, whereas rocking (10%) was less common but caused a significant impact on QoL. Non-defined balance problems were reported at 18%; these were occasional and correlated with vertigo attacks. Older participants had more frequent tripping problems. Younger participants more frequently reported *swaying and rocking*. Conclusions: Risk factors predicting poor postural control were mostly related to complaints reflecting otolith pathology. Different types of postural problems require different strategies to manage balance control and cope with the disease.

## 1. Introduction

Inner ear diseases, including hearing loss and vestibular derangements, are public health problems worldwide. One of the most debilitating inner ear disorders is Ménière’s disease (MD), which carries symptoms including hearing loss, vertigo, tinnitus, and other associated health consequences that can lead to a significantly deteriorated quality of life (QoL) [1]. Globally, the prevalence of MD in a UK Biobank study was 270 out of every 100,000 people [2]. However, a Finland study found overall disease prevalence to be at least 512 per 100,000, with an average annual incidence of 17 per 100,000 [3]. Although MD affects a relatively limited number of people in the general population, it is economically detrimental to society and individuals with MD must bear the associated burdens of significant illness [4].

While most MD patients report true vertigo, many describe their dizziness as a sensation of unsteadiness, imbalance, disequilibrium, and a feeling of unreality. Complaints such as vestibular drop attacks (VDA) with or without syncope are also reported [5]. A recent international definition of MD focuses on vertigo attacks, tinnitus, and hearing loss, but the significant impacts of the disease on gait and postural stability are less studied and not included in current disease definitions [6,7].

For postural control strategies in MD, two different stabilization mechanisms are possible: (a) a high-stiffness strategy based on the inherent elastic properties of muscles and (b) a low-stiffness strategy based on the positional feedback mechanism. The low-stiffness strategy can be implemented by means of a servomechanism and is based on explicit positional feedback from different sensory channels (e.g., proprioception and vision). However, this is an unfeasible solution due to long delays in the feedback loop, which itself becomes a source of instability. A more robust solution is to close the loop intermittently by injecting force impulses in the system through predictive control or based on sensory input [8]. However, neurophysiological studies in MD indicate that building up of vestibulospinal reflexes (VSR) is erroneous [9]. Moreover, the mechanisms controlling VSR are not able to reweigh sensory data from visual, proprioceptive, and vestibular inputs in response to changes in stimulus amplitude [10], leading to complex and often varying strategies to control balance in patients with a vestibular ailment such as MD. 

Toppila and Pyykkö [11] used a chaotic model to study body segment oscillations on a force platform—they called these “moving attractors”. This variability of attractors indicated that a postural control system can choose a new joint position in a resting state to avoid fatigue and joint overloading, and there are several neutral, energy-efficient positions that can be regarded as “attractor convenient”. High velocities or high accelerations outside areas of attractor convenience provide information on quality of control that can prevent the subject from falling. Further, Suzuki et al. [12] analyzed postural control by modelling uncontrolled manifold (UCM) and showed that, similar to Toppila and Pyykkö’s [11] attractors, the UCM component of experimental sway could be considered as passive dynamics with no active control. Suzuki et al. [12] also showed that the UCM component consists mainly of high-frequency oscillations above 1 Hz that correspond to anti-phase coordination between the ankle and hip. They indicated the close relationship between passive dynamics and intermittent control strategies, but their intermittent control hypothesis better explained the spectral characteristics of sway.

Aramaki et al. [13] measured ankle and hip joint motion during the quiet stance and reported that ranges of angular rotation, velocity, and acceleration of the hip joint angle are comparable with, or even greater than, those of the ankle joint. Moreover, they revealed that angular accelerations of both the ankle and hip joints are negatively correlated with each other at a specific ratio, suggesting that such specific coordination might reflect active control of the central nervous system in minimizing the acceleration of the center point of force on posturography. This coordination is disturbed in vestibular deprivation, as after space flight in astronauts [14]. Because postural problems create different types of sensations among individual patients, any of these different types of postural control strategies may be required. So far, however, this has not been analyzed in detail in patients with a vestibular ailment such as MD. 

Mal de Debarquement Syndrome (MdDS) has been recently defined for inclusion into the International Classification of Vestibular Disorders. This is a condition characterized by a constant feeling of swaying concentrating on 0.2 Hz [15]. Usually, MdDS begins after a boat ride or moving vehicle, but MdDS can also occur spontaneously or in connection to MD and is a maladaptation to roll movements [15]. In vestibular testing, Dai et al. [16] indicated that in MdDS there is a mismatch between otolith and semicircular canal function. They considered MdDS as a maladaptation of VOR. 

To that end, the aim of the present work was to explore the character of balance problems in MD. Patients with MD often describe poor postural stability as a “swaying sensation”, “swaying as on the deck of a ship”, “walking on cotton”, “floating”, “uncertainty of balance”, “tripping”, “constant dizziness”, or “behaving clumsily”, and so forth, without defining any characteristic of the disorder that could be classified into a specific and treatable “symptom group”. In particular, we wanted to evaluate the characteristics of balance problems to study the possibility that balance problems could partly be related to MdDS.

## 2. Method

### 2.1. Study Design and Participants

The study used a retrospective design. Permission was obtained from the Finnish Ménière Federation (FMF; Suomen Meniere-liitto) to analyze registry data the FMF had collected from their members by a questionnaire on symptoms related to MD. According to Finnish law, this study conducted by a patient organization collecting anonymous data did not require ethical approval. The FMF has 1739 members, but the electronic survey was available to only 1035 members, and from that number, 539 persons responded (i.e., 52.1% response rate) to the survey. The mean age of the study participants was 61.9 years (range 17 to 89 years). Male participants were 3.2 years older than female participants. The mean duration of the disease was 15.6 years (range 0.5 to 40 years). The respondents included 423 (i.e., 79.5%) females and 116 (i.e., 21.5%) males representing the gender distribution of FMF. In order to characterize the diagnosis of MD among FMF members, the diagnostic accuracy in a group of members (*n* = 706) was evaluated with an expert program and compared with AAO-HNS criteria [6]. Results showed that 97% of respondents had definite MD and 2.7% probable MD.

### 2.2. Data Collection

Data were gathered using an electronic questionnaire sent via the Internet. Two reminders were sent to those who did not respond to the original questionnaire. The questionnaire was evaluated by FMF board members and corrected based on their comments. The questionnaire included 36 main questions with several sub-questions. The questions were focused on socioeconomic issues and complaints of MD specifically related to balance and postural fitness, aiming to characterize any postural instability and impact caused by it. In general, the complaint-specific interference was assessed by asking the subjects to rate severity and frequency of the complaints and their impacts on a five-point scale, ranging from “none” to “very severe”. The vertigo attacks and VDA were assessed by character, provoking items, frequency, severity, and how much impact they caused. Postural stability outside the attacks, problems with gait and impairment of motility, and the impact of the balance problems were queried. Questions on the medical treatments undertaken, and the impact of tinnitus hearing loss and hyperacusis were asked. 

The survey included questions that focused on frequencies of sensation, for example: “Do you feel that you or the support surface would swing slowly (about 0.2 Hz)”, or “Do you feel that you by yourself or with the support surface would experience a rocking sensation (at about 1 Hz)?”. Frequency and intensity of balance problems and their association with visual problems were also investigated. The impact of the disorder was rated on a four-step scale from “no impact” to “severe impact”. The visual analog scale (VAS) was adopted from the EuroQol EQ-5D-3L questionnaire regarding health-related QoL questions [17]. Some of the questions were voluntary, while most of the questions were mandatory.

### 2.3. Data Analysis 

Descriptive statistics were explored. For continuous variables, Student’s *t*-test and analysis of variance (ANOVA) were performed where necessary to compare different groups. The Bonferroni post hoc test was used for pairwise comparison. Non-parametric tests, such as Chi square, Mann–Whitney U, and Kruskal–Wallis H were used for categorical variables. Stepwise regression was used in modelling the risk for binary variables. A *p*-value of 0.05 was used to interpret statistical significance. Qualitative content analysis was used to evaluate responses to open-ended questions, and the frequency of occurrence of each category was noted. 

## 3. Results

### 3.1. Prevalence of Postural and Gait Problems in MD

**Figure 1:**
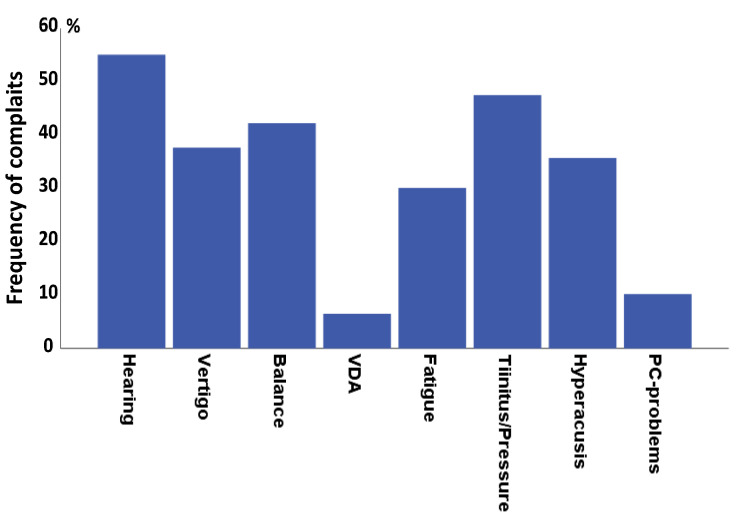
Frequently occurring complaints that impact daily life. Percentage of complaints and 95% confidence levels are shown.

Figure 1 shows major complaints that study participants reported as having a negative impact on their daily lives. Hearing problems (58%) and tinnitus/ear fullness (50%) occurred most frequently, followed by balance-related problems (44.4%). Absence of any major problems was reported by only 6.7% of the participants. It is noteworthy that vertigo (39.5%) and VDA-related (6.9%) problems were less common than balance-related problems. Hyperacusis that impairs the use of a hearing aid was present in more than one-third of the subjects (37.8%). Moreover, complaints associated with chronic MD, such as fatigue (31.7%) and PC-screen visualization problems (19.7%), were less common than balance problems. 

**Figure 2:**
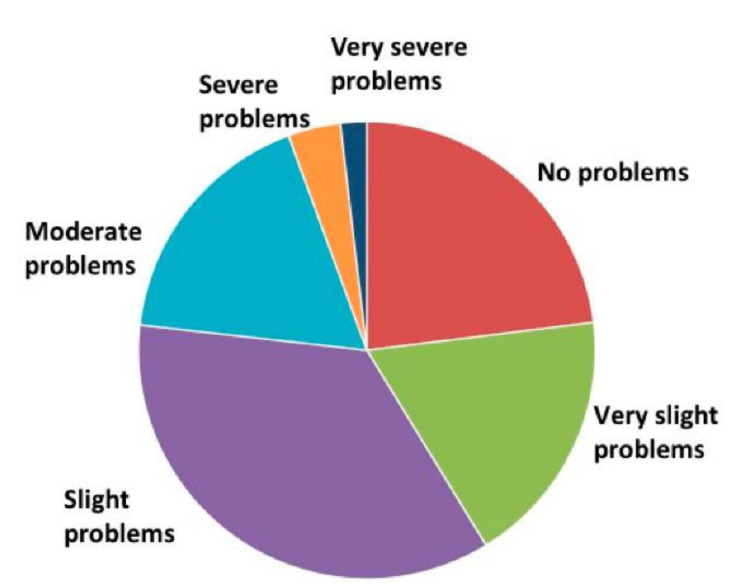
Severity of postural problems.

Out of 539 subjects with MD, 124 (23%) had not experienced any, even slight, balance problems (see Figure 2). However, the remaining subjects reported balance problems with various intensities. Most subjects had very weak (18.3%) or weak (35.6%) problems. Moderate problems (17.6%), severe problems (3.7%), or very severe problems (1.7%) were rare but had a significant impact on QoL (*F* = 89.3, *p* < 0.001).

### 3.2. Characteristics of Body Sway and Balance Problems 

**Figure 3:**
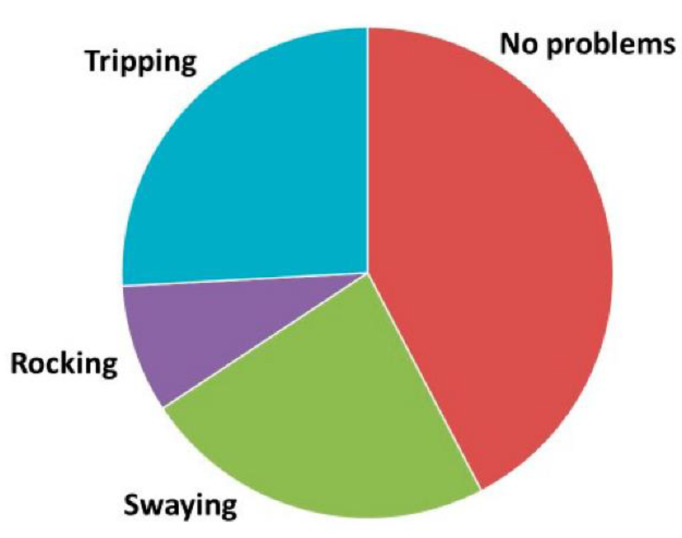
Characteristics of postural problems.

A total of 211 (i.e., 39%) participants reported no side-steps or fall tendencies. A total of 148 (27%) participants reported falling tendencies to all directions, whereas 180 (33%) reported fall tendencies to one direction only. When evaluating the characteristics of postural problems (see Figure 3), most of the subjects described swaying or a rocking sensation in their bodies, or felt that the floor was moving (30.8%). A total of 122 (22.6%) participants described slow swaying (about 0.2 Hz), whereas the rocking sensation (a faster sway, about 1 Hz) occurred less commonly (*n* = 44, 8.2%). A total of 135 (25%) participants reported tripping. Only 17 (3.2%) subjects reported combined swaying and tripping (*n* = 14) or rocking and tripping (*n* = 3). Those with non-defined balance problems described them as occasional. In the following analysis, occasional problems were grouped as no balance problems. Moreover, female participants experienced swaying-type balance problems more commonly than male participants (Mann–Whitney U-test Z = −2.6, *p* = 0.009).

**Figure 4:**
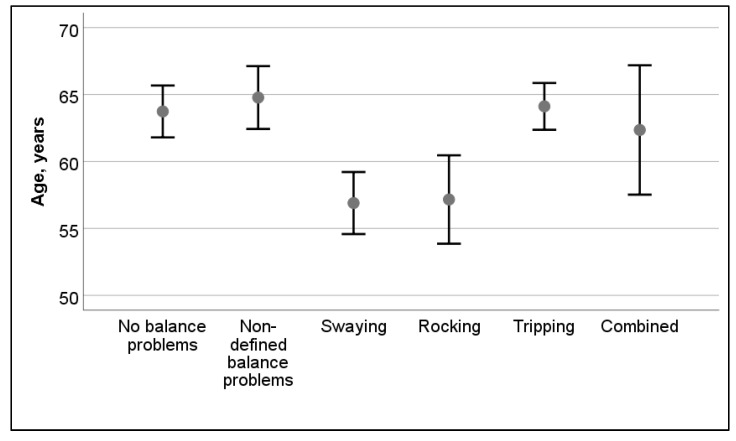
Effect of age on types of postural problems. Mean and 95% confidence interval are shown.

The age of the participant had some correlation to postural instability; in older participants, balance problems were more common (*t* = 2.7, *p* = 0.007). There was a significant difference in the types of balance problems (i.e., non-defined, swaying, rocking, tripping, or combinations of swaying, rocking, tripping) in different age groups (see Figure 4). Participants who reported swaying and rocking were significantly younger than those with no problems or those who reported tripping problems (*F* = 9.3, *p* < 0.001). In the Bonferroni post hoc test, no differences were observed between swaying and rocking, whereas both of those differed from tripping (*p* < 0.001 and *p* = 0.006, respectively). Although the same trends were observed throughout the duration of the disease, the differences between types of balance problems were not statistically significant in participants from different age groups (*F* = 1.7, *p* = 0.134). 

**Figure 5:**
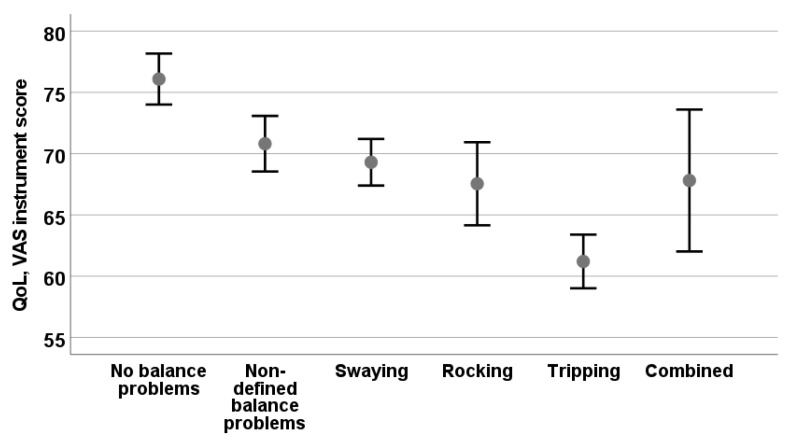
Health-related QoL measured with VAS instrument from EQ-5D-3L in groups experiencing different types of balance problems. Mean and 95% confidence levels are shown.

Considering the potential influence of a particular type of balance problem on QoL, there was a significant difference in VAS scores in participants who had no balance problems and undefined balance problems (*F* = 8.4, *p* < 0.001). In a pairwise comparison, only participants with tripping differed from baseline participants and from subjects with swinging (*p* = 0.031), but not from rocking (*p* = 0.702). Figure 5 shows the impacts of different types of balance problems on the VAS instrument. 

Regarding problems experienced while traveling in various types of vehicles, participants without balance problems had fewer issues than those with balance problems. There were noted differences in activities such as biking (H = 9.3, *p* = 0.025), driving a car (H = 15.3, *p* = 0.002), or traveling in an airplane (H = 10.9, *p* = 0.012). Moreover, all participants with balance problems considered the movement of a vehicle annoying compared to participants without balance problems, who were less likely to find that movement annoying (H = 52.9, *p* < 0.001).

### 3.3. Visual Complaints and Postural Problems

Table 1 shows the relationships of various visual complaints to the various types of balance problems. Vision was most stable in participants without balance problems (*p* = 0.004) and differed significantly from those with tripping and swaying problems. Head turn-associated visual instability was significantly different (*p* < 0.001) among the groups, as was the perception of floating objects (*p* = 0.002), focusing problems (*p* < 0.001), and problems in visualizing the horizon (*p* < 0.001). Participants with swaying problems reported the most visual complaints. 

### 3.4. Association of Balance Problems in Patients with MD 

Logistic regression analysis was performed to evaluate items explaining balance problems experienced by the subjects (see outcomes in Table 2). A statistically significant (*r^2^* = 0.155, *p* < 0.001) model was established in that nine items could predict 65% of balance problems. The variables included in the model were the age of the participants (*p* < 0.0001), *VDA* (*p* = 0.005), *fatigue* (*p* = 0.0001), *tinnitus*/pressure (*p* = 0.007), *black spots* in visual field (*p* = 0.042), *constant vertigo* (*p* = 0.002), *vertigo in attacks with constant vertigo* (*p* = 0.004), *head movement-provoked vertigo* (*p* = 0.023), and *syncope* during VDA (*p* = 0.016). Neither migraine nor headache was included in the model. Gait problems correlated significantly with balance problems (*r* = 0.409, *p* < 0.001) but were not included in the model, as gait strongly depends on balance function. 

## 4. Discussion 

The aim of the present study was to examine balance problems in individuals with MD who had a long history of ailment. Balance problems were rated as the most common complaint after hearing loss and tinnitus. The severity of their balance problems varied greatly, from very mild to very severe. In terms of impacts, most participants reported balance problems as weak (36%) or moderate (18%), whereas fewer than 5% reported severe or very severe balance problems. Balance problems were associated with various functional problems, such as fatigue, anxiety, and visual problems, and also to MD complaints, such as VDA and constant vertigo with or without vertigo attacks. The balance problems reported in this study could be classified into four main types. Tripping was the most common (34%) and significantly reduced QoL. Low-frequency swaying was present (25%) but had limited impact on QoL, whereas rocking was least common (10%) but caused a significant impact on QoL. In addition, 18% of the participants had non-specific or non-defined balance problems that were mostly temporary and associated with vertigo spells and feelings of dizziness. Those with combined rocking and tripping problems most commonly reported visual problems. Younger subjects more frequently had a combined swaying and rocking sensation than tripping or no balance problems. No significant difference was found in the duration of MD and types of balance problems. Generally, no gender differences were seen in balance problems (i.e., non-defined, rocking, tripping) between the genders, although female participants more frequently experienced swaying-type balance problems. 

Hitherto the postural problems have been medically regarded as one problem entity describing vestibular deficit in balance and gait. In the present manuscript, for the first time, we describe differences in faulty postural control mechanisms based on the patient’s own description of their problems. To our knowledge, no previous studies have examined the different characteristics of balance and gait problems in that MdDS would be atopic. We submit the hypothesis that the characteristics of swaying in MD were similar to that described in MdDS, where there is allegedly a mismatch within the otolith and semicircular canal system [16]. Rocking problems seem to be related to a low-stiffness strategy of balance control and allow for uncoordinated movements between hip and ankle joints. Tripping seems to be associated with a mismatch of vestibulospinal responses; accordingly, the subjects apply a high-stiffness strategy. Each of these postural strategies has pros and cons and may be alleviated with different types of physiotherapy or vestibular rehabilitation. In another study, we evaluate how self-training MD patients relate to their complaints [18]. The study suggested that most MD patients used self-tailored training program that aimed to alleviate their condition, especially the balance-, gait-, and VDA-associated problems. For this reason, patient organization and health care professionals should characterize the type of balance disorder and individually tailor the rehabilitation program.

### 4.1. Tripping 

Commonly in predictive conditions, muscle responses follow memorized patterns of action irrespective of whether the body is perturbed forwards or backwards [19]. In unexpected situations or after vestibular derangement, such control strategies cannot be planned and may cause balance problems. The steps become short and body movements become stiff and slow. The benefit of a stiffness strategy as implicit positional feedback is that it has an almost instantaneous response time [10]. However, stiffening the muscles consumes excessive energy and leads to fatigue. In MD, control over VSR covering segmental and proprioceptor controlling pathways is malfunctioning [9], as is vestibular memory [20,21,22]. After experiencing vestibular ailment, subjects are unable to functionally reweigh sensory data from visual, proprioceptive, and vestibular inputs in response to changes in stimulus amplitude [10].

After observing models of tripping in MD, likely due to the mismatch of inadequate VSR with postural memory, we suggest that the subject tends to select the high-stiffness strategy of the body to prevent falls. Tripping drastically impacts QoL, further stressing the importance of vestibular rehabilitation. To alleviate these types of problems, we hypothetically recommend intervention using exercises for gaze stabilization, dynamic visual acuity, dynamic and static postural stability, and desensitization to head motion. Gottshall et al. [23] set a precedent showing that this kind of training was indeed effective. 

### 4.2. Swaying

MdDS is a condition characterized by the constant feeling of swaying [15]. In the present study, 25% of participants experienced swaying, sometimes called “phantom motion” in MdDS. Usually, MdDS comes on after a ride in a boat or other moving vehicle, but MdDS can also occur spontaneously or in connection to BPPV or MD and is considered to be a maladaptation to roll movements [15,16]. Dai et al. [16] indicated that, in MdDS, there was a mismatch between otolith and semicircular canal function. Often, patients with MdDS feel better when riding in a car, boat, plane, or train. Nevertheless, in the present study, riding in vehicles did not alleviate swaying complaints and, in this respect, the findings were not typical for MdDS. MdDS is associated with many other disturbing symptoms including disorientation, impaired cognition, fatigue, ataxia, insomnia, headache, anxiety, and depression [24]. Many of these same complaints were reported in the present study and associated with postural problems. Thus, these swaying sensations correlated significantly with VDA and to visual complaints during head movements and in inspection of the horizon; thus, swaying indicates that visual and postural interaction is problematic in chronic MD and should be taken into account during patient rehabilitation. 

After observing models of swaying in MD and inspecting strategies of postural control, based on an explicit positional feedback mechanism to control their posture, we found that subjects seemed to adopt a low-stiffness strategy. Much as in MdDS, swaying in MD indicates maladaptation of VOR. For rehabilitation, we suggest optokinetic stimulation with head tilting as a therapy of choice; Dai et al. [16] developed and named this therapy “VOR Protocol”. To our knowledge, ours is the first study to suggest associations between MD and MdDS, but additional research is needed on this topic. 

### 4.3. Rocking

Toppila and Pyykkö [11] used a chaotic model to study body segment oscillations on a force platform—they called these “moving attractors”. The attractors could freely choose a new neutral, stable position when different body segments oscillated around this new attractor position. This variability of attractors indicated that a postural control system can choose a new joint position in a resting state to avoid fatigue and joint overloading, and there are several neutral, energy-efficient positions that can be regarded as “attractor convenient”. High velocities or high accelerations outside areas of attractor convenience provide information on quality of control that can prevent the subject from falling. Looking at postural problems in patients with MD, body oscillations centered around one Hz when studied on a sinusoidal moving platform [15], indicating poor central control that creates the kinds of rocking sensations reported in this study.

In this hypothesis, the passive dynamics of posture could oscillate freely until dynamic UCM intervenes and restores control to above 1 Hz. Aramaki et al. [13] suggested that uncoordinated acceleration between ankle and hip joints might reflect a loosening control of the central nervous system to minimize the acceleration of the center point of force on posturography. Vestibular-deprived subjects, such as astronauts after space flight, exhibit significant multivariate changes in multi-joint coordination, of which increased sway is only one component. These changes are consistent with the reweighting of vestibular inputs and changes in control strategy in a multivariable control system [14]. 

We hypothesize that, in MD, posture becomes unstable, and there is high oscillation between stability boundaries, and these create the rocking sensation reported here. To promote vestibular integration, visual and proprioceptive blind-walking with head movement might be useful. Moreover, while this type of therapy requires more research, we hypothetically suggest that the memorization of movement patterns paired with meditation could promote an outgrowth of the neural networks controlling posture. 

### 4.4. Study Limitations

The current study had several limitations. The study population included participants from a patient organization. The electronic survey was sent only to those who had a valid e-mail address. The response rate for the survey was low (i.e., 51%). This may have resulted in a potential sampling bias and may have possibly not included the older adults with MD who may have more frequent and/or severe postural problems. Thus, there is an uncertainty of severity and prevalence of balance problems in the MD population. Moreover, the study focused on chronic complaints and the findings may not be generalizable to newly diagnosed MD patients. 

## 5. Conclusions

The present study evaluated balance problems in patients with MD who had a long history of illness. Different types of balance problems were rated as the most common complaints after hearing loss and tinnitus. The swaying types of postural problems were found in one-third of the MD patients and were similar to those present in MdDS. The rocking types of postural problems may be related to the low-stiffness strategy of balance control and use of sensory feedback in balance control. Tripping was common and was related to poor vestibular compensation and mistrust in vestibule-spinal responses. Tripping and rocking problems significantly reduced health-related QoL and caused visual problems. Overall, the current study suggests that understanding the different types of balance problems in MD patients may assist in rehabilitation efforts by helping them select individually tailored rehabilitation programs. However, the study results should be interpreted in light of the study limitations and should be treated as preliminary. Further studies from clinical samples should examine whether the results are replicable. 

## Figures and Tables

**Table 1:** Association of visual complaints with different types of balance problems.

Character of Visual Problem	No Balance Problems (*n* = 126)	Non-Defined Balance Problems (*n* = 95)	Swaying (*n* = 122)	Rocking (*n* = 44)	Tripping (*n* = 135)	Combined Balance Problems (*n* = 17)	All (*n* = 522)	Chi-Square Test, *p*-Value
During head turn items jump/move in visual field	3 (2%)	12 (14%)	49 (40%) *^,†^	12 (27%)	30 (22%) *	4 (23%)	110 (21%)	Χ^2^ = 59.7, *p* < 0.001
Objects seem to float or move	0 (0%)	6 (6%)	14 (11%) *	11 (25%) *^,†^	7 (5%)	6 (35%)	44 (8%)	Χ^2^ = 48.3, *p* = 0.002
Problems in focusing the eyes	7 (6%)	19 (20%)	35 (29%) *^,†^	22 (50%) *	56 (41%) *	12 (70%)	151 (29%)	Χ^2^ = 72.5, *p* < 0.001
Problems in visualizing the horizon	0 (0%)	7 (7%)	29 (24%) *	9 (20%)	34 (25%) *	5 (29%)	84 (16%)	Χ^2^ = 47.1, *p* < 0.001

* Indicates differences between the groups concerned and subjects without balance problems in Mann–Whitney U-test; ^†^ indicates differences between the groups concerned and subjects with tripping in Mann–Whitney U-test.

**Table 2:** Outcomes of logistic regression analysis of complaints impacting balance problems in MD. Abbreviation: VDA = vestibular drop attack; C.I = confidence interval.

Item	Unstandardized Beta (B)	Standard Error (S.E.)	Wald	Significance	Exponentiation of the B Coefficient	95% C.I. for Exp(B) Lower	95% C.I. for Exp(B) Upper
Age	0.04	0.009	18.31	0.0001	1.04	1.02	1.06
VDA	1.19	0.42	7.985	0.005	3.29	1.44	7.50
Fatigue	0.83	0.21	15.03	0.0001	2.29	1.51	3.48
Tinnitus	0.53	0.19	7.25	0.007	1.69	1.15	2.48
Black spots	0.42	0.21	4.15	0.042	1.52	1.02	2.28
Constant vertigo	1.28	0.41	9.79	0.002	3.59	1.61	8.02
Vertigo in attacks with constant vertigo	0.82	0.29	8.29	0.004	2.27	1.30	3.98
Head movement-provoked vertigo	0.73	0.32	5.20	0.023	2.07	1.11	3.86
Syncope	1.10	0.46	5.81	0.016	3.00	1.23	7.33
Constant	−3.70	0.62	35.28	0.0001	0.025		

## Data Availability

Not applicable.
